# Supplementary material for: Real-Time Search-Assisted Acquisition on a Tribrid Mass Spectrometer Improves Coverage in Multiplexed Single-Cell Proteomics
Source: Mol Cell Proteomics. 2022 Feb 25;21(4):100219. doi: 10.1016/j.mcpro.2022.100219 (PMC8961214; doi:10.1016/j.mcpro.2022.100219)
Supplement: Supplemental Figure S1 [file mmc1.pdf]

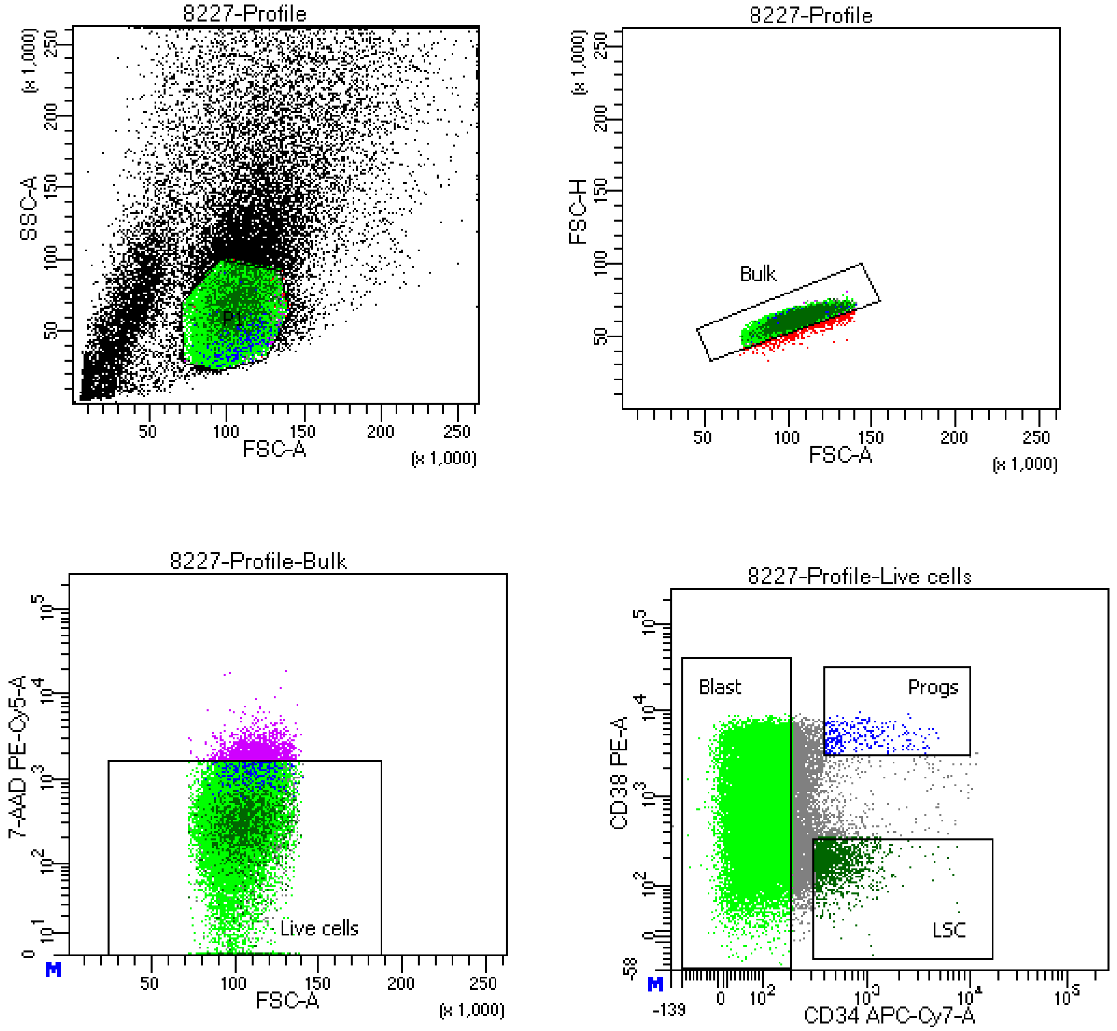

Tube: Profile

Population	#Events	%Parent	%Total
All Events	60,000	####	100.0
P1	41,198	68.7	68.7
Bulk	40,378	98.0	67.3
Live cells	38,858	96.2	64.8
Blast	32,555	83.8	54.3
Progs	284	0.7	0.5
LSC	1,337	3.4	2.2

Experiment Name:	8227 single cell sort_07Jun...
Specimen Name:	8227
Tube Name:	Profile
Record Date:	Jun 7, 2021 11:37:30 AM
$OP:	Administrator
GUID:	2877c1ee-60e8-4cc3-96ac...

Population	#Events	%Parent
Live cells	38,858	96.2
Blast	32,555	83.8
Progs	284	0.7
LSC	1,337	3.4

Supplementary Figure 1. FACS strategy for single-cell sort of OCI-AML8227 cell-culture system.
