# Supplementary material for: Real-Time Search-Assisted Acquisition on a Tribrid Mass Spectrometer Improves Coverage in Multiplexed Single-Cell Proteomics
Source: Mol Cell Proteomics. 2022 Feb 25;21(4):100219. doi: 10.1016/j.mcpro.2022.100219 (PMC8961214; doi:10.1016/j.mcpro.2022.100219)
Supplement: Supplemental Figure S2 [file mmc2.pdf]

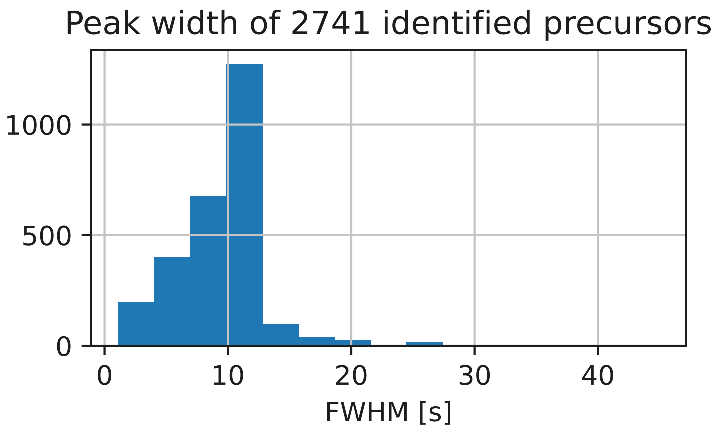

Supplementary Figure 2. Chromatographic peak widths observed in our LC setup. Full width at half maximum (FWHM) in seconds of 2,741 precursors identified in one run of MS2 500ms. Median is 10.43 s. FWHM was calculated using apQuant.
