# Supplementary material for: Real-Time Search-Assisted Acquisition on a Tribrid Mass Spectrometer Improves Coverage in Multiplexed Single-Cell Proteomics
Source: Mol Cell Proteomics. 2022 Feb 25;21(4):100219. doi: 10.1016/j.mcpro.2022.100219 (PMC8961214; doi:10.1016/j.mcpro.2022.100219)
Supplement: Supplemental Figure S3 [file mmc3.pdf]

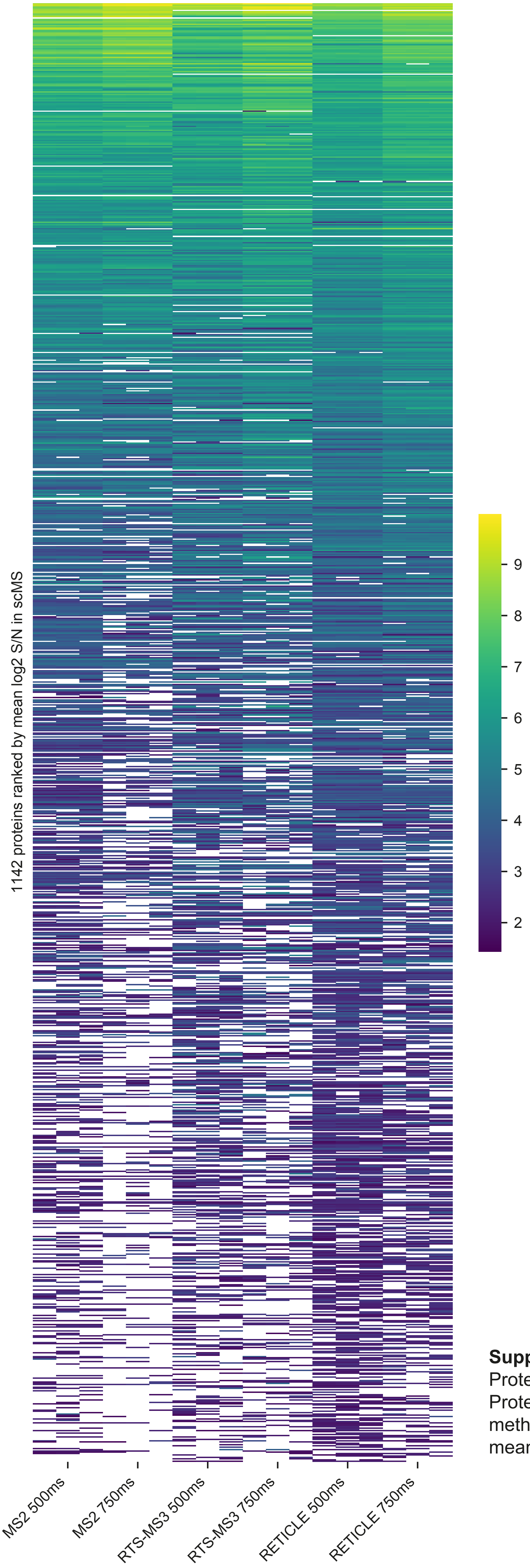

Supplementary Figure 3. Full heatmap version of Figure 3A. Protein expression matrix of each method measured in triplicates. Proteins in the rows were sorted by the mean log2 S/N across all methods and each individual row shows the same protein with its mean log2 S/N across all cells in each LC-MS run.
