# Supplementary material for: Real-Time Search-Assisted Acquisition on a Tribrid Mass Spectrometer Improves Coverage in Multiplexed Single-Cell Proteomics
Source: Mol Cell Proteomics. 2022 Feb 25;21(4):100219. doi: 10.1016/j.mcpro.2022.100219 (PMC8961214; doi:10.1016/j.mcpro.2022.100219)
Supplement: Supplemental Figure S4 [file mmc4.pdf]

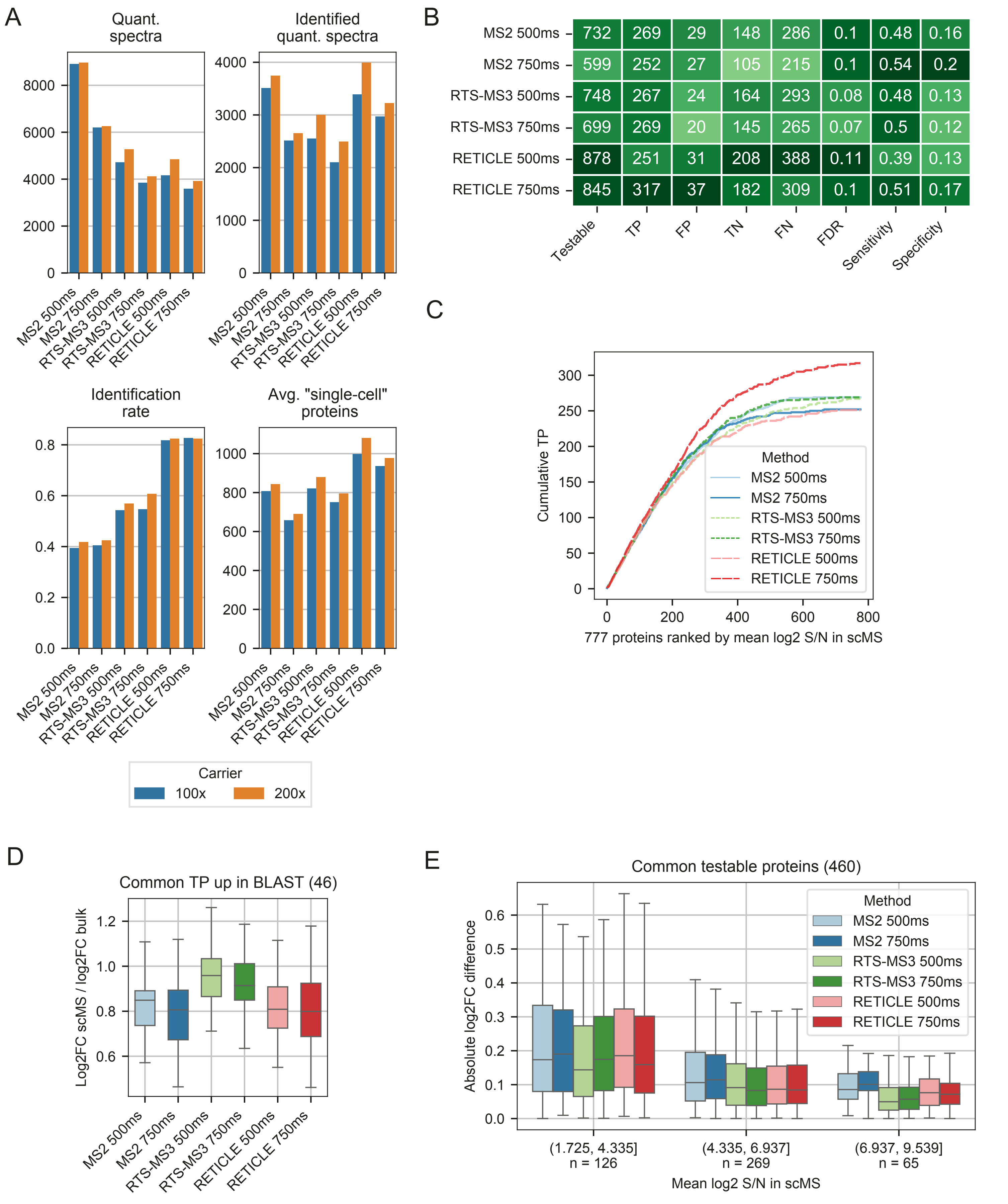

Supplementary Figure 4. Evaluation of a 100-cell carrier. A) Comparison of spectra acquisition, their identification and the resulting proteome coverage between 100x and 200x samples. B) Differential protein expression analysis of the 100x data between BLAST and LSC of each method using the bulk-measured reference dataset for validation. TP=true positive, FP=false positive, TN=true negative, FN=false negative (see Methods). C) Cumulative distribution of true positive DE proteins of the 100x data ordered by mean log2 S/N across all methods. Proteins on the x-axis are the same for each method and represent the union of all testable proteins across all methods that are DE in the bulk-measured reference. D) Ratio compression in the 100x data measured by dividing log2FC in scMS by log2FC in the bulk-measured reference. Common true positive proteins that were upregulated in BLASTS were used. Outlier not shown. E) Absolute log2FC difference between 100x scMS data and the bulk-measured reference. The intersection of testable proteins across all methods was used and binned by mean log2 S/N across all methods. Outlier not shown.
