# Supplementary material for: Real-Time Search-Assisted Acquisition on a Tribrid Mass Spectrometer Improves Coverage in Multiplexed Single-Cell Proteomics
Source: Mol Cell Proteomics. 2022 Feb 25;21(4):100219. doi: 10.1016/j.mcpro.2022.100219 (PMC8961214; doi:10.1016/j.mcpro.2022.100219)
Supplement: Supplemental Figure S5 [file mmc5.pdf]

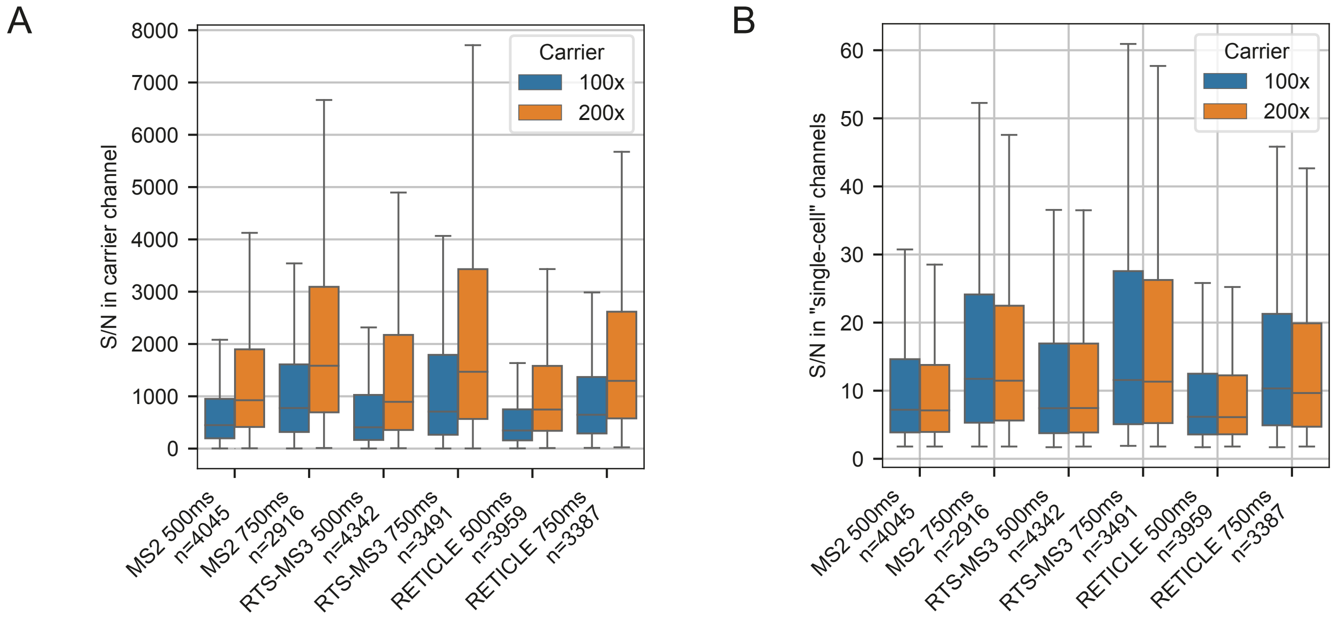

Supplementary Figure 5. Comparison of peptide S/N values on PSM level between 100x and 200x carrier samples. A) Mean S/N of peptides in carrier channel for each method and carrier amount. Mean was calculated from the PSMs of the same peptide from the three replicates of each method. Only peptides that were overlapping between both carrier levels were used. B) Mean S/N of peptides in the “single-cell” channels from the same PSMs as in A. Outliers not shown.
